# Obituary for Dr Harriet Nabudere (1973–2021), Former Managing Editor of the East African Health Research Journal

**DOI:** 10.24248/eahrj.v5i2.659

**Published:** 2021-11-15

**Authors:** Fabian M. Mashauri, Novat Twungubumwe, Sam Okware

**Affiliations:** a East African Health Research Commission, Bujumbura, Burundi; b Uganda National Health Research Organization, Kampala, Uganda

**Figure:**
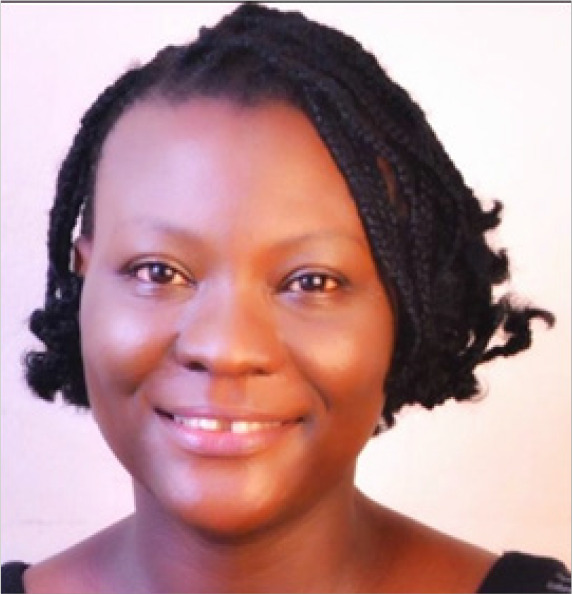


It is with great sadness we learned about the sudden demise of our colleague, and former Managing Editor of the East African Health Research Journal (EAHRJ) Dr Harriet Nabudere who passed away on 07^th^ August 2021. Dr Nabudere is remembered as one of the founders of the EAHRJ. Dr Nabudere was instrumental for establishing the infrastructure of the EAHRJ notable the journal Editorial Manager (EM) system. Her range of contributions on publishing EAHRJ issues regularly was extraordinary. Dr Nabudere initially was an associate editor of the EAHRJ before she was promoted to the position of Managing Editor (ME).

Dr Nabudere also was the East African Health Research Commission (EAHRC) National Focal Point (NFP) Coordinator representing the Republic of Uganda, since the inception of the EAHRC 2015. She had coordinated the EAHRC activities in Uganda diligently by linking the Commission with the National Stakeholders. By doing so she significantly contributed to making the EAHRC known in the Republic of Uganda.

We remember Dr Nabudere for her outstandingly successful editorship and mentorship to the many young scientists in the region and advancing knowledge dissemination in the East African Community (EAC) region. She will be missed by many friends, the EAHRC, Uganda National Health Research Organization (UNHRO), authors as well as reviewers for the EAHRJ. She was a research manager and supported the development and evaluation of knowledge translation strategies for Health systems with European and Developing Countries Clinical Trials Partnership (EDCTP) between 2009 and 2014

Dr. Nabudere studied at Makerere University, Uganda from 1992 to 1998 where she obtained MBChB. She proceeded with postgraduate studies at the BRAC University of Dhaka, Bangladesh where she obtained a Master's degree in Public Health in 2006. She had 15 years' experience as a Public Health specialist in knowledge management and translation. She had skills in research synthesis and developing policy formats for policy makers in low resource settings. She undertook several consultancies works in the EAC region providing technical support in training and mentoring of health workers.

Her research encompassed a broad range of area including research synthesis where research evidence from health research was acquired, assessed for quality, and packaged in user-friendly formats for policy makers and decision-makers to be adapted for application in low-resource settings. This included development and evaluation of research or knowledge synthesis products and services; such as evidence-based policy briefs and policy dialogues, to engage multiple national stakeholders within Uganda's health system and applicable across Low Middle Income Countries (LMIC) contexts. She authored and published several articles which has been cited worldwide.

One of the distinct hallmarks of her life will be her innate intellectual generosity and her ability to emotionally connect with her colleagues, academicians, and scientific collaborators, and her unselfishness in her support of people from different cultural environment and social attitudes.

Dr Harriet Nabudere is survived with a daughter Esther Mukite.

On behalf of all Editors, Associate Editors, and Editorial Board Members who had the privilege to work with or know Dr Harriet Nabudere, Our deepest condolences to the family.

